# Bridging the gap between GLP1-receptor agonists and cardiovascular outcomes: evidence for the role of tirzepatide

**DOI:** 10.1186/s12933-024-02319-7

**Published:** 2024-07-10

**Authors:** Fatemeh Taktaz, Rosaria Anna Fontanella, Lucia Scisciola, Ada Pesapane, Manuela Giovanna Basilicata, Puja Ghosh, Martina Franzese, Giovanni Tortorella, Armando Puocci, Maria Teresa Vietri, Annalisa Capuano, Giuseppe Paolisso, Michelangela Barbieri

**Affiliations:** 1 Department of Advanced Medical and Surgical Sciences, University of Campania “Luigi Vanvitelli”, Naples, Italy; 2 Department of Precision Medicine, University of Campania “Luigi Vanvitelli”, Naples, Italy; 3 Clinical and Molecular Pathology, A.O.U. University of Campania “Luigi Vanvitelli”, Naples, Italy; 4 Department of Experimental Medicine, University of Campania “Luigi Vanvitelli”, Naples, Italy; 5 UniCamillus, International Medical University, Rome, Italy

**Keywords:** Tirzepatide, GLP-1 receptor, GIP receptor, GLP1 receptor agonists, Heart failure

## Abstract

Tirzepatide is a new drug targeting glucagon-like peptide 1(GLP1) and gastric inhibitory polypeptide (GIP) receptors. This drug has demonstrated great potential in improving the clinical outcomes of patients with type 2 diabetes. It can lead to weight loss, better glycemic control, and reduced cardiometabolic risk factors. GLP1 receptor agonists have been proven effective antidiabetic medications with possible cardiovascular benefits. Even though they have been proven to reduce the risk of major adverse cardiovascular events, their effectiveness in treating heart failure is unknown. Unlike traditional GLP1 receptor agonists, tirzepatide is more selective for the GIP receptor, resulting in a more balanced activation of these receptors. This review article discusses the possible mechanisms tirzepatide may use to improve cardiovascular health. That includes the anti-inflammatory effect, the ability to reduce cell death and promote autophagy, and also its indirect effects through blood pressure, obesity, and glucose/lipid metabolism. Additionally, tirzepatide may benefit atherosclerosis and lower the risk of major adverse cardiac events. Currently, clinical trials are underway to evaluate the safety and efficacy of tirzepatide in patients with heart failure. Overall, tirzepatide’s dual agonism of GLP1 and GIP receptors appears to provide encouraging cardiovascular benefits beyond glycemic control, offering a potential new therapeutic option for treating cardiovascular diseases and heart failure.

**Graphical abstract:**

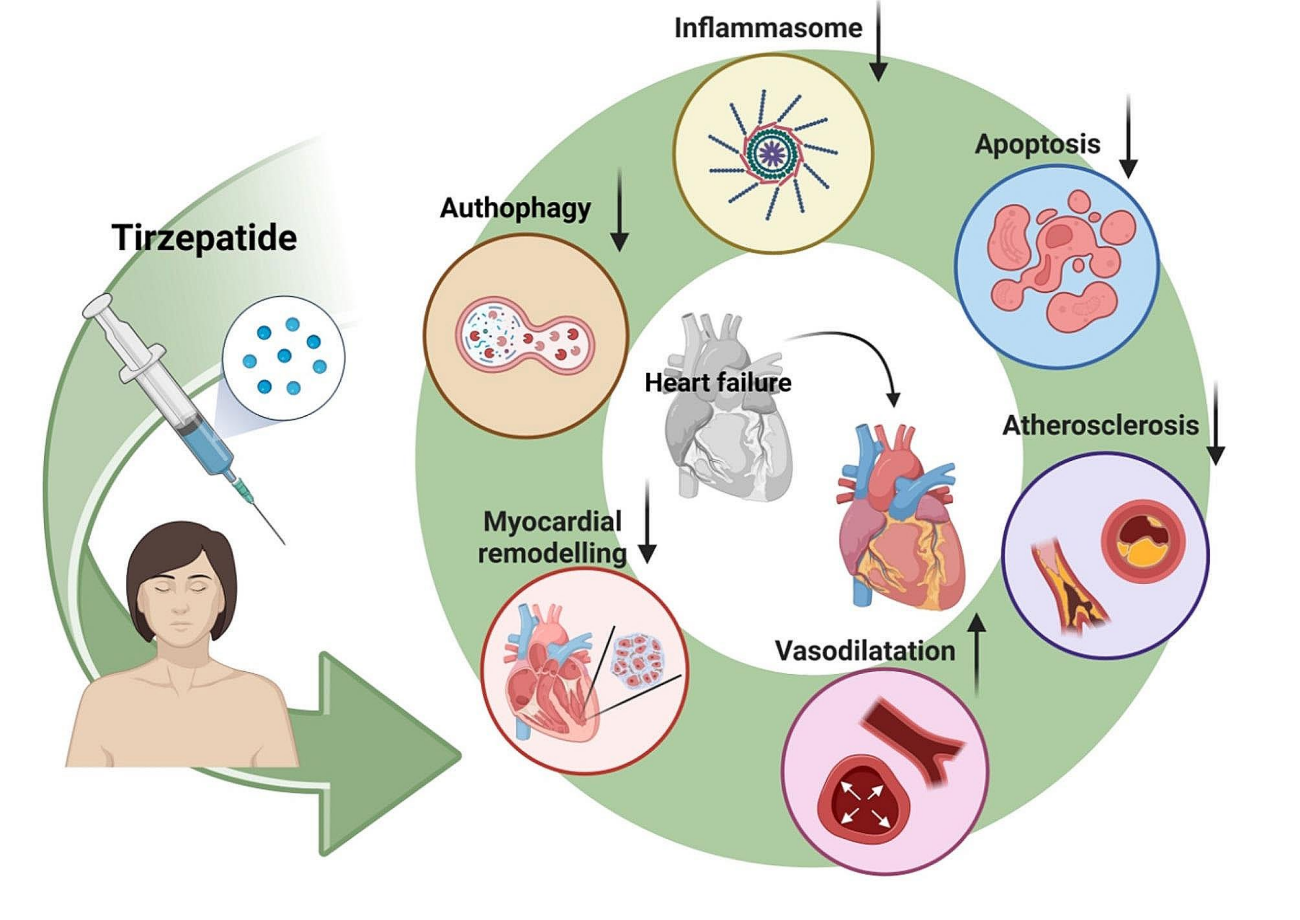

## Introduction

Tirzepatide (LY3298176) is the first peptide dual agonist for type 2 diabetes mellitus (T2DM), which targets both GIPR and GLP1R. According to primary trials in T2DM, tirzepatide significantly reduces clinical outcomes like body weight, glycated hemoglobin (HbA1c), lipid profiles, and glucose-adjusted glucagon secretion compared to typical glucagon-like peptide1 receptor agonists (GLP1RAs) [1, 2]. Additionally, new research indicates that tirzepatide potentially has some advantages over traditional metabolic regulation, such as insulin sensitivity improvement and anti-inflammatory effect [2]. The dual mechanism of tirzepatide has a synergistic impact that is strong enough to significantly lower the risk of cardiovascular events [3, 4]. Studies suggest tirzepatide may help improve cardiometabolic risk factors and reduce the risk of atherosclerotic cardiovascular disease (CVD). These risk factors include blood pressure, cholesterol, and chronic inflammation [3]. In SURPASS 4 The risk of suffering from any major adverse cardiovascular event MACE-4 and MACE-3 (including myocardial infarction, stroke, hospitalization for angina, and all-cause death) with the highest dose of tirzepatide (15 mg per week) has been estimated at 0·50 (0·26–0·95), which was intended to provide initial proof of the cardiovascular safety of tirzepatide [2, 5]. The FIGHT trial specifically evaluated using the GLP-1RAs, liraglutide, in patients with HF with reduced ejection fraction (HFrEF). Although the trial did not show a significant advantage in reducing cardiovascular events [6], or hospitalizations, it provided insights into liraglutide’s safety and tolerability in this patient population [6]. Ongoing research, such as the SUMMIT trial, is presently assessing the safety and efficacy of tirzepatide in HF patients with preserved ejection fraction (HFpEF). However, comprehensive data on the direct effects of HF are still being developed (NCT04847557). Additionally, the positive outcomes of ongoing trials SURPASS-CVOT and SURMOUNT-MMO will provide definitive evidence regarding the cardiovascular safety and efficacy of tirzepatide compared to dulaglutide and reduction in morbiditiy and mortality in adults with obesity respectively. CVOT trial will help determine if tirzepatide can demonstrate superior cardiovascular safety and potential additional cardiovascular benefits compared to the currently approved GLP-1RA dulaglutide. the data from these two large, well-powered clinical trials will provide robust evidence on the cardiovascular and metabolic benefits of tirzepatide, potentially expanding the treatment options for adults with T2DM, obesity, and related cardiovascular complications [7] (Clinical Traials.gov). SURMOUNT-1 trial suggests that tirzepatide may have a promising future as a therapy for obesity, HF, and non-alcoholic fatty liver disease (NAFLD) [5, 8]. Previous meta-analyses have indicated that tirzepatide might potentially decrease the risk of cardiovascular events by affecting cardiac cells, the pancreas, and other tissues directly and indirectly [2]. Also, the efficacy and safety of tirzepatide may have a wider perspective for its use beyond patients with T2DM or obesity [9, 10]. This review will discuss the limitation of GLP1RAs in patients with HF, along with ongoing trials assessing the safety and effectiveness of tirzepatide, look into the existing data, and elucidate tirzepatides potential impact on cardiovascular disease.

## The role of GLP-1/GIP receptors: tirzepatide imbalanced and biased activation

Human atrial and ventricular cardiomyocytes express the GLP1R within each of all chambers, and the sinoatrial (SA) node shows the highest level of GLP1R expression [11, 12]. GIPR, unlike the GLP1R, is extensively expressed in the ventricular myocardium of both rodents and humans. Boosting GIPR activity could directly affect the myocardium and indirectly influence cardiovascular function and outcomes through its systemic effects on other organs [13]. Identifying GLP1R and GIPR in endothelial cells, vascular smooth muscle cells, and blood has underscored their significant roles in vascular function [11]. The activation of GLP1R and GIPR by specific ligands triggers intracellular signaling cascades that regulate the structure and function of the heart. Both receptors canonical signaling pathway involves coupling to Gαs proteins, and in turn, triggers adenylyl cyclase activation and leads to the production of cyclic adenosine monophosphate (cAMP). The increased cAMP levels in cardiac cells activate exchange protein directly activated by cAMP (EPAC) and protein kinase A (PKA), which regulate a multitude of downstream effectors involved in cardiac contractility, relaxation, and hypertrophy [14]. and GIP receptors have also been demonstrated to activate phosphoinositide 3-kinase (PI3K) and its downstream effector Akt, which modulates mitochondrial function, metabolism, and cell survival. Recent data indicates that both receptors may interact through β-arrestin-dependent pathways, potentially contributing to their cardioprotective properties without involving G protein signaling [15]. Interestingly, the rise in hormone-sensitive lipase (HSL) phosphorylation within cardiac myocytes appears to rely on a GIPR-mediated increase in ERK phosphorylation. This implies that GIP activity may regulate myocardial triacylglycerol (TAG) metabolism. Additionally, the infusion of native human GIP (1–42) into cardiac myocytes isolated from embryonic mouse hearts inhibited the increase in brain natriuretic peptide (BNP) and transforming growth factor- β (TGF- β 1) mRNA expression induced by angiotensin II (Ang II) [13]. This could be the key to tirzepatide’s remarkable efficacy. It is expected that tirzepatide will occupy a greater percentage of GIPR than GLP1R (4.1, 4.6, and 6.3 fold, respectively, at 15, 10, and 5 mg) [16]. Tirzepatide is formulated to mimic the function of native GIP at the GIPR while also interacting with the GLP1R in a manner different from native GLP1. Additionally, tirzepatide was designed to bind to the GIPR with an affinity 5-fold stronger than that for the GLP1R, resulting in greater potency and selectivity in engaging the GIPR over the GLP1R. Also, experiments conducted in primary islet cells have shown that β-arrestin1 restricts the insulin secretion response to GLP1R activation, but not to GIPR activation or tirzepatide. This indicates that the biased agonism properties of tirzepatide, which preferentially activates specific signaling pathways, enhance its ability to stimulate insulin secretion [17, 18]. Additionally, tirzepatide demonstrates bias at the GLP1R, favoring the synthesis of cAMP over the recruitment of β-arrestin [1]. Studies have found that tirzepatide’s hormone-secreting activities depend on GIPR, highlighting the critical role that GIPR plays in the medication’s mechanism of action and its therapeutic effects [19]. Tirzepatide appears to activate GIPR stronger than GLP1R in cell systems with high levels of both gene expressions [20]. Tirzepatide has three critical improvements over first-generation GLP1RAs. Firstly, it alters multiple residues in the peptide structure to mimic GIP role at GIPR. Additionally, it extends the C-terminal with a sequence of exenatide, and thirdly, it has an attached fatty acid side chain, similar to semaglutide, to prolong its half-life [16].

### Tirzepatide and cardiovascular disease

A previous study has shown that tirzepatide can protect human cardiomyocytes by effectively ameliorating apoptosis, fibrosis, and hypertrophy when exposed to high glucose. This highlights tirzepatide’s potential as an alternative treatment for clinical outcomes related to HF management. However, further research is needed to evaluate its impact on HF outcomes in clinical settings [21].

## Atherosclerosis

Up to 85% of cardiovascular deaths are related to atherosclerotic heart disease, which occurs as heart attacks and strokes. In the cardiovascular system, atherosclerosis becomes apparent as the “invisible killer.” Atherosclerosis causes cerebral ischemia, or angina pectoris, with the development of atherosclerotic plaques. This may result in severe clinical manifestations like myocardial infarction (MI) and HF [22–24]. In the LEADER trial, liraglutide demonstrated cardiovascular benefit, and GLP1RAs have similar effects, aligning with liraglutide’s anti-atherosclerotic effect. GLP1RAs have shown the ability to decrease the development and advancement of atherosclerosis in preclinical and clinical investigations [25, 26]. There is also a current clinical study examining injectable semaglutide’s effect on coronary atherosclerosis development [27]. Moreover, a decrease in atherosclerotic cardiovascular disease (ASCVD) has been found in people treated with semaglutide who were obese or overweight, but, this trend was not statistically significant [28]. Based on SURMOUNT-1 data analysis, tirzepatide decreased the 10-year estimated risk of ASCVD for obese or overweight patients. It has been shown that tirzepatide can lower the risk of ASCVD by improving lipid, glucose, and HbA1c levels, as well as blood pressure and waist circumference. Interestingly enough, patients with higher baseline ASCVD risk with tirzepatide therapy showed significantly more reductions in risk [3]. It is crucial to address ASCVD disease in T2DM at an earlier stage, with more intensively and precision. Several factors contribute to the heightened risk of atherosclerosis, and a comprehensive approach is essential for preventing or reducing atherosclerotic events[29]. Preclinical studies have indicated that GLP1RAs, such as tirzepatide, may directly impact the vasculature by enhancing endothelial function and decreasing inflammation, which are important in preventing atherosclerosis[30]. Tirzepatide has the potential to prevent atherosclerosis risk in T2DM patients without pre-existing lesions. For those with established atherosclerosis, the focus would be on managing the existing condition and preventing further progression [31]. Tirzepatide has been shown in clinical trials to reduce the risk of cardiovascular events at various doses, suggesting a potential treatment option for patients who are obese or overweight [2]. According to the study conducted by Nagashima et al., treatment of 17-week-old mice with GIP via subcutaneous injection showed a significant decrease in the size of aortic atherosclerotic lesions and macrophage infiltration. These findings emphasize the importance of GIPR activity and its anti-sclerotic role [32–34]. Moreover, according to research by Ojima et al., a GIP concentration of 50 pM effectively suppressed the generation of reactive oxygen species (ROS) in human umbilical vein endothelial cells (HUVECs) exposed to AGE. A reduction in the expression levels of the AGE receptor facilitated this effect. The study also noted a decrease in the expression level of pro-atherogenic molecule factors such as plasminogen activator inhibitor-1 (Pai-1) [35]. GIP may reduce oxidative stress in endothelial cells and visceral adipose tissue and decrease the secretion of inflammatory cytokines [36]. Moreover, activation of endothelial nitric oxide synthase (NOS) in HUVECs by GIP at 1 nM decreased inflammatory inducible NOS expression levels. It increased the production of NO, which is an anti-atherogenic molecule [37, 38]. Moreover, GIP at pharmaceutical doses may prevent atherosclerosis, causing obesity, and the positive benefits of GIP may also be maintained in animals [39]. Tirzepatide, with a dual activity target GIP and GLP1 receptor, may have consequences for preventing or mitigating atherosclerosis due to its impact on GIPR activity.

## Major adverse cardiovascular events (MACE)

The research examined eight large cohorts of T2DM patients, with cardiovascular outcome trials (CVOTs), for main MACE outcomes, including cardiovascular death, nonfatal MI, and nonfatal stroke, which were studied with GLP1RAs [40]. The primary and secondary outcome endpoints of MACE and nonfatal stroke demonstrated significant reductions in the dulaglutide-treated REWIND trial and the subcutaneous semaglutide-treated SUSTAIN-6 study [41]. Recent evidence has suggested that GLP1RAs may decrease the incidence of stroke in people with T2DM. To evaluate the incidence of MACE-4 events in patients treated with tirzepatide compared to the control group, a meta-analysis was conducted using data from 7778 patients enrolled in the SURPASS-4 study, SURPASS Clinical Trails Program (which includes 7 randomized clinical trials), and SURMOUNT-1. The overall hazard ratio (HR) estimate was 0.59 (95% CI 0.40–0.79), indicating that tirzepatide resulted from a significant reduction in the risk of MACE-4 compared to the control group [21]. As tirzepatide was compared to controls, the hazard ratios for MACE-4 were 0.80 (95% confidence interval [CI], 0.57–1.11), cardiovascular death was 0.90 (95% CI, 0.50–1.61), and all-cause death was 0.80 (95% CI, 0.51–1.25) [2]. It is essential to keep in mind that the current data suggests tirzepatide may be efficient in downgrading the possibility of stroke; however, trials developed mainly to evaluate the long-term impact of tirzepatide on cardiovascular events, including stroke, are currently in progress [42].

## Heart failure

Heart failure is a prevalent disease that impacts nearly 2% of the global population. Diagnosis of HF requires the presence of an underlying cardiac abnormality. Usually, this is a cardiac abnormality that results in either diastolic or systolic ventricular dysfunction (e.g., myocardial infarction or coronary artery disease). Moreover, damage or weakness in the heart muscle itself, disorders of the valves such as stenosis or regurgitation, endocardium, cardiac conduction and rhythm, pericardium, or all of these changes can cause HF [43, 44]. Also, both systolic and diastolic dysfunction stemming from the interplay between atherosclerotic coronary disease and diabetic cardiomyopathy are associated with the underlying pathophysiology of HF in patients with T2DM [45, 46]. Several mechanisms may contribute to the positive effects of GLP1RAs in preventing HF and influencing the outcomes for patients with either HFpEF or HFrEF. Also GLP1RAs have been found to decrease epicardial fat, which may help explain their potential in lowering the risk of atherosclerosis. On the other hand the GLP1R in both cardiomyocytes and sinoatrial node cells can activate cyclic AMP (cAMP)-dependent signaling pathways. This may lead to an excessive influx of calcium into cells. This calcium dysregulation could be associated with the pathophysiology of HF in patients with diabetes [47]. Sardu et al. found that GLP1RAs significantly raised LVEF and 6-minute walking test (6MWT) in T2DM with HF [48]. According to previous studies, liraglutide attenuates myocardial hypertrophy and fibrosis following angiotensin II infusion by suppressing TGF-β1/Smads signaling pathways [49]. Nevertheless, GLP1RAs did not statistically significantly improve systolic function, as demonstrated by Kumarathurai et al. [50]. The findings revealed that GLP1RAs could extend 6MWT and lower the risk of HF hospitalization in people without a history of HF; however, their effects on HF hospitalization with a history of HF, NT-proBNP levels, and quality of life were not statistically significant. GLP1RAs significantly decreased E-wave velocity, E/A ratio, E/e _ ratio, and EDT but did not affect LVEF. In addition, patients with HF can have their cardiac function and prognosis assessed using 6MWT, NT-proBNP, and QoL [51]. A single trial that evaluated GLP1RAs in patients with a history of HF and HFpEF demonstrated reduced HF hospitalizations. However, this trial was limited by small sample size and potential flaws in the selection of endpoints (NCT04916470 and NCT05371496) [52]. The SUMMIT study assesses tirzepatide’s impact on HF with HFpEF in obese patients. The research suggests that for patients with HF with HFrEF, the dual therapy of tirzepatide and guideline-directed medical therapy (GDMT) may be a viable treatment approach. GDMT for HFrEF typically includes evidence-based beta-blockers, angiotensin receptor neprilysin inhibitors (ARNIs), angiotensin receptor blockers (ARBs), angiotensin-converting enzyme (ACE) inhibitors, mineralocorticoid receptor antagonists (MRA), and sodium-glucose cotransporter 2 inhibitors (SGLT2is), particularly in patients with obesity or T2DM [53]. Despite the lack of research on tirzepatide, especially in HF patients, its cardiovascular effects indicate potential benefits for this population. Tirzepatide is presently being examined in a clinical trial (NCT04847557) for patients with moderate HF (NYHA class II-IV) with HFpEF [54]. The research outcomes suggest that human cardiac cells treatment with tirzepatide resulted in an augmentation of Sarco/Endoplasmic Reticulum Calcium ATPase 2 (SERCA2) and p-Phospholamban (p-PLN) activity and expression while reducing Calcium/Calmodulin-Dependent ProtienKinase II (CAMKII) expression. These markers are pivotal in regulating calcium signaling, and the intervention effectively mitigates calcium overload in cardiomyocytes exposed to elevated glucose levels. Furthermore, the study showed that tirzepatide correlated with a marked decrease in the levels of key indicators associated with fibrosis and hypertrophy, such as TGF- β, Matrix Metalloprotieinase-9 (MMP9), Collagen Type I Alpha 1 (COLIA1) and F-box only protein 32 (FBXO32) [21].

### Direct mechanisms of tirzepatide on cardiovascular health

Although the precise mechanism by which tirzepatide could benefit HF is not known, several potential pathways have been suggested:

### Anti-inflammatory effects

Patients with T2DM experience increased levels of hyperinsulinemia, hyperglycemia, and insulin resistance. These conditions result in the overproduction of AGEs, accumulation of pro-inflammatory and pro-fibrotic factors, and free fatty acid oxidation. These outcomes lead to the remodeling of the extracellular matrix, oxidative stress, inflammation, cardiomyocyte apoptosis, fibrosis, and metabolic abnormalities [55]. It has been determined that inflammation has been identified as a severe risk factor for the development of cardiovascular diseases [56]. Excessive ROS production can lead to cellular damage by increasing the formation of AGEs, its receptor (RAGE), protein kinase C (PKC), and NF-κB. GLP1RAs can inhibit neutral lipid accumulation, ROS generation, and (NADPH oxidase 4) NOX-4 expression in rat cardiomyocytes, thereby migrating interleukin-1 β (IL-1 β) induced ROS production [57]. After 26 weeks of tirzepatide treatment at different doses, several biomarkers associated with inflammation were reduced. These biomarkers include leptin, chitinase-3 like-protein-1 (YKL-40), growth differentiation factor 15 (GDF-15), and intercellular adhesion molecule 1 (ICAM-1). These circulating markers are linked to inflammation and endothelial dysfunction, which can lead to cardiovascular events. C-reactive protein (CRP) and YKL-40 concentrations were lower after tirzepatide administration compared to the baseline. Additionally, tirzepatide treatment decreased the levels of both markers compared to placebo and dulaglutide [31]. Combining tirzepatide with GLP-1 and GIP hormones may lead to a more significant reduction in inflammation compared to GLP1RAs alone due to their anti-inflammatory effects [58]. Recent research has shown that GIP helps reduce oxidative stress in human endothelial cells and prevents the release of inflammatory cytokines in visceral adipose tissue [59]. Liu et al. concluded that tirzepatide was found to be effective in improving cardiac dysfunction and increasing survival rates in mice that were treated with LPS. The study revealed that tirzepatide can reduce inflammation and apoptosis caused by LPS in the heart. This suggests that tirzepatide inhibits the activation of TLR4/NLRP3/ NF-κB inflammasome signaling pathways. The study also found that tirzepatide reduces the risk of ventricular arrhythmia in LPS-treated mice [60]. It is generally accepted that TLR4 plays an essential role in cardiac inflammation, and NF-κB serves as a downstream mediator of TLR4 in the heart. Previous research has shown that the NLRP3 pathway is one of NF-κB’s downstream pathways [61–63]. Ventricular arrhythmias are typical of critically patients suffering from acute sepsis and have been linked to poor outcomes. According to a study by Chuang et al., exposure to LPS increases this population’s risk of ventricular arrhythmias [64, 65]. Previous research has shown that GLP1 (9–36) can decrease levels of IL-1 β, interleukin 6 (IL-6), and tumor necrosis factor-α (TNF-α). Another research has evaluated that blocking NLRP3-mediated inflammation and apoptosis can reduce cardiac damage caused by LPS [66]. A study found that tirzepatide treatment can reduce inflammation in the hearts of sepsis mice. This suggests that tirzepatide may have cardioprotective benefits by acting as an anti-inflammatory agent [67]. Furthermore, tirzepatide therapy in diabetic rats restored specific abnormal alterations in signal molecules related to inflammatory signaling pathways via PI3K/Akt/GSK3β signaling pathway [68].

### Cell death and autophagy

**Fig. 1:**
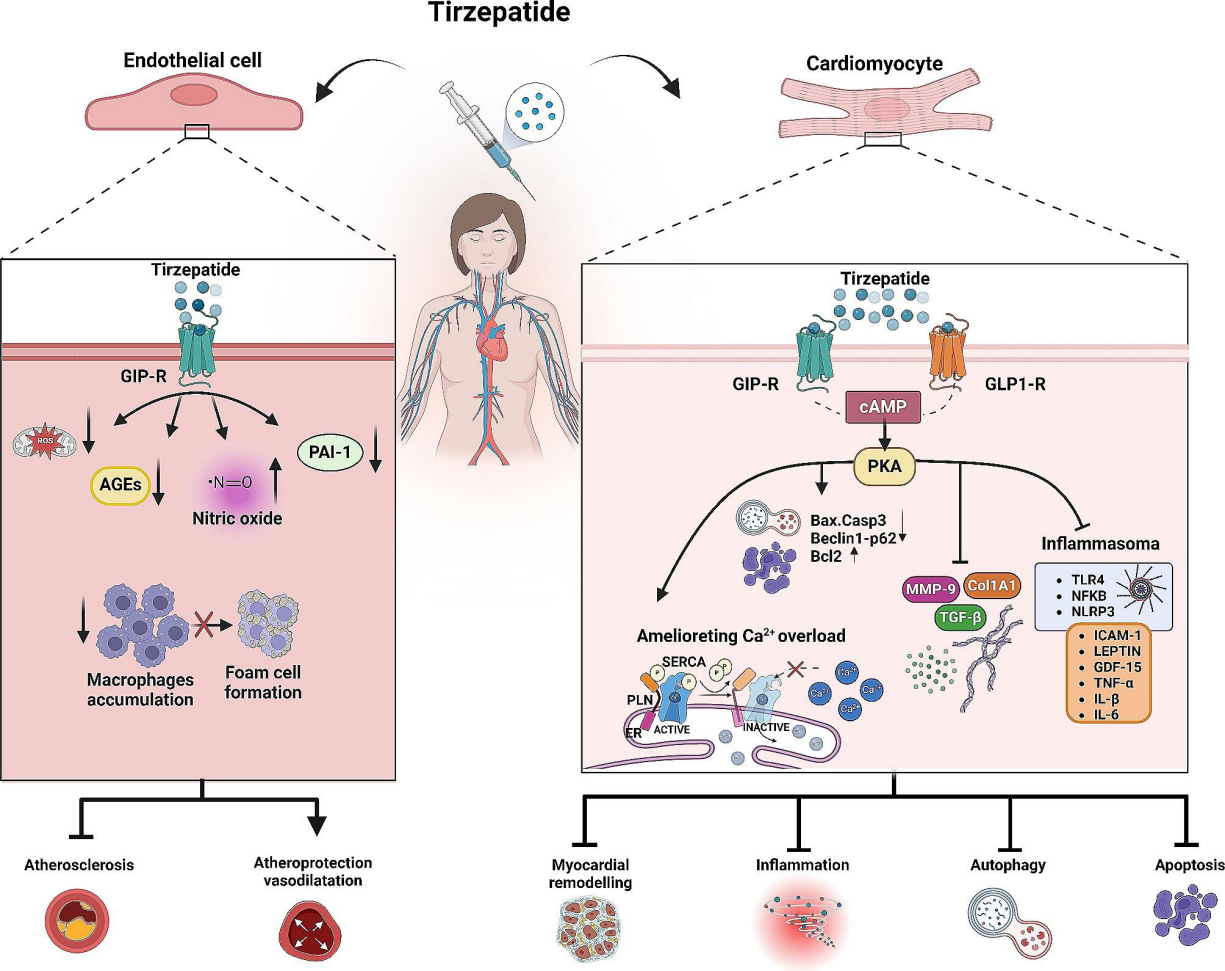
Comprehensive picture of molecular and cellular interaction relevant to beneficial effect of tirzepatide in metabolic and cardiovascular disorders. It is signaling pathways associated with GIP and GLP1 receptors, as well as key molecules cAMP and PKA involved in cellular processes related to cardiovascular pathophysiology. The diagram highlights an in-depth understanding of the complex network of pathways that influence conditions like atherosclerotic, inflammatory response, and metabolic dysregulation

Mitochondria, the powerhouse of cells, require proper maintenance through processes like mitophagy, fusion, fission, and biogenesis to perform their physiological functions. However, in diabetic cardiomyopathy, there is a disruption in the quality control of mitochondria, which results in an inadequate fusion process, increased fission, and fragmentation [69]. Although only a small number of cardiomyocytes experience cell death due to apoptosis, activating apoptotic pathways can worsen cardiac dysfunction. Therefore, apoptosis is frequently associated with the mechanisms that cause sepsis-induced cardiac depression [70–72]. In our previous studies, tirzepatide therapy elevated the levels of anti-apoptotic marker Bcl2 and reduced the levels of Bax in human cardiac cells. Additionally, it significantly reduced the activation of CASP3, a crucial component of apoptotic pathways. It also led to a significant reduction in the autophagy biomarkers Beclin1 and p62, which indicates an increase in autophagy flux [21] Fig. 1.

### Tirzepatide’s indirect impact on cardiovascular system

#### Glucose and lipid metabolism

The study conducted by Karagiannis et al. found that tirzepatide led to significant dose-dependent reductions in HbA1c levels when compared to a placebo, once-weekly GLP-1RAs, and basal insulin regimens. Interestingly, there was no correlation between the positive effect on glycemic control and an increased risk of hypoglycemia [73]. Tirzepatide’s insulinotropic effects on human islets depend on GIPR activity, according to Ei et al. [19]. It is possible to hypothesize that activation of GIPR has the potential to protect from ischemic heart, given its similar effects on insulin production and myocardial Akt phosphorylation in mice and also a cardioprotective effect of ligargudide further supports this hypothesis [74]. It is suggested that activating GIPR can increase the development of white adipose tissue (WAT), which can enhance long-term lipid storage and improve adipocytes’ ability to remove dietary triglycerides (TAG) [75, 76]. Also, GIP is a substance that enhances insulin sensitivity in adipocytes. It may increase glucose uptake by breaking down TAGs in chylomicrons through LPL activity and transporting glucose through a protein called GLUT4. These effects seem to be mediated by GLUT4, which triggers PI3K/PKB, and glucose, which inhibits the LKB1/AMPK pathway. Additionally, pCREB, nuclear translocation, and transcriptional activity of TORC2, a protein complex, also play a role in LPL recruitment. These mechanisms are different from the classical signaling pathway of GIPR [75]. Tirzepatide is a medication that improves insulin sensitivity, which can be evaluated indirectly through fasting biomarkers such as glucose and insulin and the homeostasis model assessment of insulin resistance (HOMA2-IR). When compared to GLP1RAs, which improve insulin sensitivity primarily through weight reduction, a post-doc regression analysis of HOMA2-IR indicates that only 20–30% of the improvement in insulin sensitization observed with tirzepatide is due to weight loss (NCT03951753) [77, 78]. In poorly managed T2DM treated with basal insulin, tirzepatide can improve glucose control without hypoglycemia risk, alone or in combination with other oral hypoglycemic medications [79]. A recent clinical trial has found that in patients with T2DM, tirzepatide performed better than GLP1RAs and dulaglutide monotherapy regarding glycaemic management and body weight reduction. In the same trial, tirzepatide and semaglutide were compared once weekly in the open-label, phase III SURPASS-2 experiment. Regarding average changes in HbA1c levels, tirzepatide demonstrated both non-inferiority and superiority to semaglutide at all doses [80, 81]. HbA1c is crucial for assessing diabetes severity and HF development risk [82]. Tirzepatide may improve glycemic control by directly and indirectly affecting the pancreas and other tissues. It increases insulin secretion from pancreatic β-cells, reduces glucose-adjusted glucagon secretion, and improves insulin sensitivity more than what can be typically achieved through weight loss. This means that tirzepatide has the potential to improve glycemic control better than GLP1RAs [77, 83]. Studies such as SURPASS 1–5 and J-mono have demonstrated that tirzepatide is more effective than other therapy alternatives in reducing HbA1C levels [84, 85]. In a study conducted on Japanese patients with T2DM, those who received tirzepatide doses ranging from 5 to 15 mg for 52 weeks experienced a significant decrease in HbA1c levels by 2.4–3.02%. Furthermore, compared to dulaglutide, tirzepatide at a dose of 0.75 mg once weekly was more effective at controlling blood glucose levels in this cohort. In all SURPASS programs, tirzepatide demonstrated a much greater HbA1c-lowering impact than placebo or active comparators, such as basal insulin and GLP1RAs [83, 86]. Over 250 patients underwent metabolomics and lipidomic investigations, which revealed that unlike the baseline and placebo, tirzepatide, taken over 26 weeks, significantly impacted a group of lipids and metabolites like 3-hydroxybutyrate reduced branched-chain ketoacids (BCKA), branched-chain amino acids (BCAA), and direct catabolic products linked with obesity and insulin resistance. It is worth noting that the changes observed in HbA1c, proinsulin levels, and indication of insulin resistance were more pronounced with tirzepatide than with dulaglutide. This suggests that tirzepatide is more effective in improving glucose control in patients with T2DM [87, 88]. Tirzepatide has been observed to decrease the levels of low-density lipoprotein particles (LDLP) and massive low-density lipoprotein particles (TRLP) over time and also can reduce triglyceride, apoB, and apoC-III levels dose-dependently. The most significant changes were observed at the highest dosages of tirzepatide, i.e., 10 and 15 mg [89, 90]. Directly activating GIPR in hepatocytes is an unlikely explanation for the decrease in apoC-III levels with tirzepatide [91, 92]. Tirzepatide is a promising drug for patients with T2DM who also have hypertension and hyperlipidemia. It has been found to increase the concentration of HDL (high-density lipoprotein) while decreasing the concentration of VLDL (very low-density lipoprotein) and triglycerides. Moreover, a recent SURPASS-3 MRI substudy has shown that tirzepatide may be more effective in reducing liver fat than insulin degludec [93].

### Obesity and blood pressure

Several GLP-1RAs, such as liraglutide, lixsenatide, semaglutide, dulaglutide, and exenatide, are utilized in clinical outcomes for the treatment of T2DM as well as obesity [94]. Liraglutide and semaglutide are GLP1RAs licensed for treating obesity (BMI > 30 kg/m²) and overweight people with comorbidities [95]. Also, Liraglutide treatment reduced blood pressure (BP) and plasma lipid levels among SCALE weight-loss program participants [96]. The primary outcome measure for the SELECT trial is the incidence of three-point MACE. SELECT CVOT trial investigated the cardiovascular safety of semaglutide 2.4 mg therapy once a week in a group of 17,605 overweight or obese adults over the age of 45 with no history of T2DM. [97]. Furthermore, GIPR antagonists increase metabolism in obese mice, and when combined with GLP1RAs, both GIPR agonists and antagonists have beneficial therapeutic effects. Also, GLP1/GIP receptors antibody fused protein was previously found to improve body weight loss [98]. Compared to dulaglutide, tirzepatide at both 5 and 10 mg doses has shown improved glycemic control as well as weight management with similar levels of tolerability. This suggests that the GLP1/GIP pathway can increase efficacy over GLP-1RAs [99]. Tirzepatide was found to be effective in promoting insulin secretion dependent on glucose through GIPR and GLP1R activation in both in vivo and in vitro models. The therapy led to weight loss in a mouse model of obesity by decreasing food intake and increasing energy expenditure [100]. Tirzepatide is a medication that can help people lose weight by slowing down food movement from the stomach to the small intestine and reducing appetite. As obesity is known to increase the risk of cardiovascular disease, including HF, therefore, anti-obesity interventions like tirzepatide may have cardioprotective effects [101]. Additionally, the activation of GIP may amplify the weight loss and insulin secretion effects of GLP1 activation in the central nervous system and pancreas, respectively. GIP activation may also improve lipid processing and growth of white adipose tissue, resulting in complementary processes. However, GIPR agonism can potentially decrease the nauseous effects of GLP1 agonism by inhibiting area postrema inhibitory neurons. This could allow higher therapeutic doses of GLP1 analogs to be administrated with fewer side effects [75]. Based on the SURPASS-2 trial, tirzepatide, when taken once a week in doses of 10 or 15 mg, is more effective in reducing blood pressure than semaglutide, which is taken once a week in a dose of 1 mg. The difference in effectiveness could be attributed to tirzepatide’s GIP agonism and its more robust ability to promote weight loss. The mean systolic blood pressure reduction was 5.3 mm Hg for semaglutide, 6.5 mm Hg for tirzepatide 15 mg, and 3.6 mm Hg for tirzepatide 10 mg. It is believed that the blood pressure reduction caused by tirzepatide results from various factors such as weight loss, vasodilation, natriuresis, concurrent medication, and reduction in extracellular volume [102]. Tirzepatide has been shown to positively affect blood pressure, possibly due to improved endothelial function or decreased inflammation. A post hoc analysis of the phase IIb trial showed that tirzepatide was associated with a reduction in the level of high-sensitivity ICAM-1, CRP, and YKL-40 after 26 weeks of treatment in a dose-dependent manner [83, 103]. A phase 3 study conducted on patients with T2DM has shown that tirzepatide improves HbA1c levels at all doses (5–15 mg). Tirzepatide is twice as effective as semaglutide in average weight loss after a 40-week treatment period (– 12.4 kg compared to– 6.2 kg) [8]. According to the SURPASS program, which included five studies, researchers reported that a weekly dose of tirzepatide at 15 mg significantly reduced systolic blood pressure by as much as 12.6 mm Hg [97]. By contrast, GLP-1RAs dosing over the long term may only slightly increase systolic blood pressure by 2 mmHg [104].

### Vasodilatory effect

Studies showed that GIPR and GLP1R are present in the smooth muscle cells and endothelial arterioles and may have a cardioprotective effect due to endothelium-dependent vasodilation [105]. In a study comprising 16 patients with T2DM and 12 non-diabetic controls, flow-mediated vasodilation was enhanced in both groups by GLP1 infusion of 0.4 pmol/kg/min, administered during a 2-hour hyperglycemic clamp [106]. It is currently unclear whether GLP1RAs resistant to degradation produce the same effects on blood flow as native GLP1. In control experiments, both native GLP1 and GLP1(9–36) have been shown to produce vasodilation and increase cGMP release from isolated, preconstricted blood vessels when tested ex vivo. On the other hand, exenatide did not affect endothelial function in rat conduit arteries when tested ex vivo after intralipid infusion [107, 108]. It has been proposed that GIP regulates inflammation and leukocyte adhesion through endothelin 1 while promoting vasodilation via nitric oxide secretion [109]. Researchers have discovered that GIP is crucial in regulating vascular and cardiac function and lipid metabolism. GIP influences not only endothelial cells but also adipose tissue and circulating triglycerides, which suggests that it may significantly impact the cardiovascular system. Studies have shown that variations in the GIPR gene are linked to cardiovascular disease and metabolic syndrome characteristics, which supports this idea [110]. Tirzepatide has the potential benefit of improving metabolic control and cardiovascular function because of its dual activity on GIP and GLP-1 receptors. Further research is required to comprehensively understand tirzepatides impact on vasodilation and its precise effects on cardiovascular function.

### Common adverse events and side effects

Tirzepatide may cause common side effects in irritable bowel syndrome, such as diarrhea, constipation, nausea, vomiting, upper abdominal discomfort, decreased appetite, and abdominal pain. This might suggest that tirzepatide affects the gut microbiota [81]. Numerous gastrointestinal conditions, such as irritable bowel syndrome, inflammatory bowel disease, and small intestinal bacterial overgrowth, have been associated with dysbiosis (an imbalance or disruption in the composition and diversity of the gut microbiome) [111]. According to research, modifying the gut microbiota can impact various physiological functions, including cognitive behavior and muscle performance [112]. The potential relationship between tirzepatide and the gut microbiome is not fully understood, making it an appealing area of study. Understanding these interactions could provide insight into the mechanisms behind tirzepatide’s metabolic benefits and inform future strategies for improving its efficacy and minimizing side effects [113]. During the phase 3 trial of tirzepatide, 6.6% of patients discontinued the medication due to side effects [114]. Furthermore, pancreatitis or cholelithiasis patients should use tirzepatide with caution [84].

## Conclusion

A new drug named tirzepatide has been approved to treat T2DM, and it is expected to be approved for weight loss by activating both GLP-1 and GIP receptors through a unique mechanism. Currently, there are other dual GIPR/GLP-1R agonists available, such as GLP-1R/GR (glucagon receptor), GLP-1R/AR (amylin receptor), and GLP-1R/NPYR (peptide YY binds to neuropeptide Y receptors) [36, 115] and even triple agonists that can activate GIPR to treat obesity and T2DM. This has led to renewed interest in understanding the safety of GIP/GIPR in humans. It is essential to gain a deeper understanding of GIP safety to guide future research projects and develop GIP-based treatments [116]. The post hoc analysis found that tirzepatide at all doses investigated reduced the prevalence of metabolic syndrome related to cardiovascular risk factors more than placebo, semaglutide 1 mg, insulin degludec, and insulin glargine [117]. Comparing tirzepatide to GLP1RAs using the results of prior comparison trials is challenging. However, the added agonism of GIPR seems to have a more positive impact on weight loss. It is important to develop a single pharmacological medication with multiple functions, such as tirzepatide. This medication can increase insulin sensitivity, decrease weight, and treat dyslipidemia at an early clinical stage, while also dramatically lowering blood sugar levels. Therefore, it seems that tirzepatide is more than just a new antidiabetic drug. The development of further dual GLP-1/GIP receptor agonists seems to be a promising next step in managing various cardiovascular disease. However, further research is needed to determine the long-term impacts of these substances. The role of tirzepatide in therapy will become more evident as more research is conducted.

## Data Availability

No datasets were generated or analysed during the current study.

## Abbreviations

T2DM: Type 2 Diabetes Mellitus
GLP1: Glucagon-like peptide1
GIP: Glucose-dependent insulinotropic polypeptide
HbA1c: Hemoglobin A1c
GLP1RAs: Glucagon-like peptide1 receptor agonists
GIPR: Glucose-dependent insulinotropic polypeptide receptor
GLP1R: Glucagon-like peptide1 receptor
CVD: Cardiovascular Disease
MACE: Major Adverse Cardiovascular Event
HF: Heart Failure
HFrEF: Heart Failure with Reduced Ejection Fraction
HFpEF: Heart Failure with Preserved Ejection Fraction
ASCVD: Atherosclerotic Cardiovascular Disease
CVOTs: Cardiovascular Outcome Trials
SA: Sinoatrial
ICAM-1: Intercellular Adhesion Molecule 1
GDF15: Growth Differentiation Factor 15
CRP: C-reactive protein
LPS: Lipopolysaccharide
TLR4: Toll-like receptor 4
NLRP3: NOD-like receptor protein 3
TNF-α: Tumor Necrosis Factor-alpha
IL-1β: Interleukin-1 beta
IL-6: Interleukin-6
TUNEL: Terminal deoxynucleotidyl transferase dUTP nick end labeling
BCAA: Branched-chain amino acids
BCKA: Branched-chain ketoacids
HOMA2-IR: Homeostasis Model Assessment of Insulin Resistance
LDL: Low-Density Lipoprotein
VLDL: Very Low-Density Lipoprotein
BP: Blood Pressure
